# Original Antigenic Sin and Respiratory Syncytial Virus Vaccines

**DOI:** 10.3390/vaccines7030107

**Published:** 2019-09-06

**Authors:** Ralph A. Tripp, Ultan F. Power

**Affiliations:** 1 Department of Infectious Diseases, University of Georgia, Athens, GA 30602, USA; 2 Wellcome-Wolfson Institute for Experimental Medicine, School of Medicine, Dentistry and Biomedical Sciences, Queen’s University Belfast, Belfast BT9 7BL, Northern Ireland

**Keywords:** original antigenic sin, OAS, RSV, vaccines, vaccine effectiveness, VE, immunological memory

## Abstract

The original antigenic sin (OAS) theory considers the outcome of the first encounter with an antigen. It favors a memory response to the original antigen upon exposure to a similar or related antigen, and includes both positive and negative impacts of past exposure on the memory response to challenge, and, in particular, on vaccine efficacy. This phenomenon is closely linked with imprinting and the hierarchical nature of immune responses to previously encountered antigens. The focus of this commentary centers on the potential role of OAS or immunological imprinting on respiratory syncytial virus memory responses.

Original antigenic sin (OAS) is the propensity of the immune response to favor responses to an original antigen over a related or new antigen. OAS typically causes immunologic memory to be more robust to the original antigen relative to new antigens, so a hierarchal position in the response occurs over time. This may lead to a moderated response during successive antigen experience and diminished vaccine effectiveness (VE) as senior antigenic responses become focused in the immune repertoire and each subsequent antigenic exposure leads to a lessened response. This does not imply that OAS leads to impaired responses, only that OAS can affect the original memory response to the original infecting strain or vaccine antigen. This may be relevant to the immune response to respiratory syncytial virus (RSV), a major cause of acute lower respiratory disease in infants and young children, often resulting in hospitalization and substantial morbidity, particularly in developing countries [1,2]. RSV causes repeated acute respiratory infections in young children accounting for > 33 million cases annually, with nearly 10% of patients resulting in hospitalization with a mortality rate around 1%–3% of those hospitalized [2,3]. RSV also causes substantial morbidity and mortality in the elderly and immunocompromised individuals [2,3].

Vaccines for prevention of RSV are clearly needed. RSV vaccine development continues to be challenging despite ongoing efforts for more than five decades. Various factors have contributed to the delay in RSV vaccine and therapeutic development [2,4,5,6,7,8]. However, the main setback in RSV vaccine development occurred in the 1960s, when a formalin-inactivated alum-precipitated RSV vaccine (FI-RSV) was injected intramuscularly in RSV-naïve infants, and following natural RSV infection of vaccinees, a large proportion developed enhanced respiratory disease (ERD) [9,10,11]. The ERD manifested itself clinically as pneumonia, bronchiolitis, rhinitis, or bronchitis. Postmortem examination of two vaccinees who died following RSV infection revealed bronchopneumonia with emphysema and pneumothorax, and histological examination of their lung tissues demonstrated a massive infiltration of monocytes, neutrophils, and, to a lesser extent, eosinophils [12,13]. Sera from the FI-RSV vaccines were found to have reasonably high anti-RSV F protein and low anti-RSV G protein antibody titers and poor in vitro neutralizing antibody titers [14]. The RSV-naïve children who received the FI-RSV vaccine may have developed OAS with consequences related to RSV pathogenicity. This contrasted starkly with older FI-RSV vaccinees who were infected with RSV prior to FI-RSV vaccination and in whom no ERD was reported. As such, the RSV-specific OAS for these older children was natural RSV infection. Therefore, the type and perhaps administration route of the original RSV antigen influence the immune responses to and clinical outcomes of subsequent RSV infections. This FI-RSV-induced ERD phenomenon resulted in a de facto moratorium on the subsequent development of inactivated or subunit RSV vaccines for RSV-naïve infants.

FI-RSV-induced ERD has been reproduced to some extent in some small animal models of RSV infection. For example, several studies have demonstrated ERD following RSV challenge of FI-RSV-vaccinated mice and cotton rats or mice scarified with recombinant vaccinia virus expressing RSV G, whereas vaccination with other RSV vaccine candidates, such as BBG2Na, recombinant Sendai virus expressing RSV F, or priming with live RSV, did not induce ERD following RSV challenge [15,16,17,18,19,20,21]. Evidence suggests that the severity of FI-RSV-induced ERD may be diminished when formulated with the TLR4 agonist monophosphoryl lipid A [22]. This is consistent with studies that demonstrated that vaccination with inactivated influenza virus PR8 strain, formulated in either pertussis toxin or a squalene-based oil-in-water nanoemulsion, prevented OAS in subsequent immunization with the closely related FM1 strain [23].

Currently, around 60 RSV vaccine candidates are in various stages of development ranging from preclinical to Phase 3 studies [1,5,24,25]. The vaccine candidates include particle-based, subunit-based, and gene-based vector approaches that are designed to elicit broadly protective and safe immune responses [26]. Unfortunately, all RSV vaccine candidates tested to date have failed to progress beyond Phase 2 or failed to meet their primary endpoints in Phase 3. This was the case for a recent Phase 3 clinical trial examining the Novavax RSV F protein maternal vaccine, which failed to prevent medically-significant RSV-mediated lower respiratory tract infections, joining the other failed F protein vaccine candidates trialed to date [1,3,27].

In general, RSV vaccine candidates can be classed into three platform technologies: protein-based, gene delivery, and live-attenuated vaccines. The protein-based stratagems include subunit vaccines (e.g., pre- and post-fusion F protein, G protein, F/G proteins, or peptides), whereas the gene delivery approaches include nucleic acid vaccines, and replication-competent or deficient vectors such as adenovirus, modified vaccinia Ankara (MVA), or Sendai virus-vectored vaccines having RSV antigen gene insertions. Perhaps the greatest efforts toward developing RSV vaccine candidates has been centered on developing live-attenuated RSV vaccines as the ERD risk has been demonstrated to be minimal or non-existent and live-attenuated vaccines resemble natural RSV infection [5,7,28]. The target populations for RSV vaccines are those at the extremes of age (the very young and the elderly), those that are immune compromised, and pregnant women [3,5,24,29]. Infants less than six months of age are at the highest risk of severe RSV disease due to the waning of maternal antibodies. Children six months or older are targeted for reasons similar to infants but this population has a more mature immune system. Pregnant women are targeted to induce high levels of neutralizing antibodies that will be transferred to the fetus and protect the infant as well as block RSV transmission. Finally, the elderly are targeted as they often have waning immunity [10]. Our current understanding dictates that the types of RSV vaccine candidates to be used will be dependent upon age, health, and immune status of the recipient, as no universal vaccine approach is currently being considered for RSV. Thus, the type of vaccine used will depend upon the level of reactogenicity, and, in the case of live-attenuated candidates, the level of attenuation. As all vaccine responses are shaped by immune experience and imprinting, and as all adults have experienced multiple RSV infections with many different RSV strains, evaluating VE in such populations will be difficult as antibody responses will reflect established memory and may not be predictive of VE and protective immunity. It is also important to consider that the immune response to a RSV vaccine is intrinsically different in RSV-naïve infants than other age groups due to their relative immunological immaturity and lack of previous RSV experience and immunological imprinting.

To prevent RSV infection in the most susceptible population, i.e., infants, one solution is maternal vaccination. This involves passive protection through immunization of pregnant mothers in the third trimester where maternal antibodies cross the placenta and protect babies through the first months of life. This approach is currently used for influenza virus protection [30]. As the peak incidence of RSV-induced LRTI occurs between six weeks and six months of age, vaccine-induced protection of young infants may be preferred but remains problematic because young infants have immature immune systems. As such, vaccination is likely to be most effective in older infants who have likely experienced RSV. Notably, all mothers are RSV experienced and have a level of pre-existing humoral immunity that does not provide complete protection from re-infection; thus, they can be repeatedly infected with RSV. However, maternal vaccination combined with pre-existing RSV immunity could aid VE in the infant and perhaps increase the longevity of protective responses. An efficacious maternal RSV vaccine would seem to be one that stimulates elevated antibody titers given pre-existing immunity, reducing viral replication, leading to protection and reduced disease. As no safe and effective RSV vaccine remains available, it is unclear if maternal vaccination influences OAS in infants. The presence of maternal antibodies that fail to protect from infection can provide insights into the role of antibody responses that are needed for VE.

Antibodies to the RSV F protein may neutralize RSV in vitro but not fully protect from disease [1,31,32]. An example is the disappointing Phase 3 clinical trial assessing a Novavax RSV F protein maternal vaccine [27]. Likewise, the results from immune prophylaxis studies in high-risk patients with monoclonal antibodies to the RSV F protein (palivizumab) showed that administration is effective at reducing hospitalization but is not fully protective against disease [1,33,34,35]. Structural differences between pre- and post-fusion F protein likely have a role in RSV vaccine outcome and OAS. Pre-fusion F protein is absent on the surface of FI-RSV [36], whereas infectious RSV has both pre- and post-fusion F conformations. This antigenic distinction is likely linked to VE. The significance of RSV F protein antigenic regions and immune dominance in the development of OAS is becoming better defined [37], which will aid in our understanding as cellular and antibody-mediated immunity contribute to differences in OAS. Other approaches are available to induce immunity to RSV such as targeting the G protein. Consistent with the need to study maternal RSV vaccines, a recombinant protein, BBG2Na, which contains a RSV G protein fragment (amino acid (aa) 130–230) including conserved amino acid residues (aa 164–176) fused to the albumin-binding region of streptococcal protein G, was shown to induce substantial protective efficacy following challenge of BBG2Na-vaccinated BALB/c mice and cotton rats against both homologous and heterologous RSV challenge [38,39,40]. BBG2Na-induced protective immunity was maintained even after early life immunization in the presence of high titers of RSV- or BBG2Na-induced maternal antibodies [41]. The protective efficacy was shown to reflect the high capacity of the vaccine to escape inhibition by RSV-induced maternal antibodies. Thus, immunization with BBG2Na elicited anti-G protein antibodies that protected against viral challenge despite neonatal immunologic immaturity and the presence of maternal anti-RSV antibodies.

Vaccines are fundamental public health tools, but they do not confer complete protection due to a variety of features often linked to variation in immunogenicity and host responsiveness [42,43]. VE varies by age as well as with vaccination or infection history, consequences driven by antigen imprinting. The OAS model proposes that the original antigen experience shapes the immune response that is boosted upon subsequent re-exposure to the same or similar antigens and an abridged antibody response to novel antigens [44]. Studies have shown reduced influenza VE year-to-year and reduced VE in repeat vaccines, regardless of age, despite high anti-hemagglutinin (HA) titers toward any historic immunizing flu strain [44,45]. The results showing activation of influenza-specific memory B cells upon sequential antigen exposure suggest that antigen relatedness, but not necessarily the duration between antigen exposures, may be a significant factor regarding VE [33,34]. The results also suggest that non-neutralizing antibodies that allow for re-exposure may contribute to the diversification of the memory B cell pool and benefit protection against evolving influenza virus strains [35].

OAS is fundamentally important in vaccine development because a vaccine strategy that stimulates immune diversification could change the VE upon the appearance of a variant strain. As noted, RSV causes repeat infections which implies that RSV does not induce durable and robust immunity, and the humoral and cellular immune responses to RSV are known to be insufficient [42,43,44]. Repeat RSV infections may be analogous to suboptimal vaccination, and in the context of OAS, levels of restricted immunity may modify the immune repertoire and RSV memory response. Age and host genetics affect the balance of immune responses, and studies examining RSV infection of the young infant have shown that the age at initial infection has a role in determining the severity of disease [46,47]. How OAS affects the original response to the infecting strain and subsequent infections needs to be better understood, particularly in RSV vaccine development.
